# When less is more: hormesis against stress and disease

**DOI:** 10.15698/mic2014.05.148

**Published:** 2014-05-05

**Authors:** Andreas Zimmermann, Maria A. Bauer, Guido Kroemer, Frank Madeo, Didac Carmona-Gutierrez

**Affiliations:** 1 Institute of Molecular Biosciences, University of Graz, 8010 Graz, Austria.; 2 Equipe 11 Labellisée Ligue Contre le Cancer, INSERM U1138, Centre de Recherche des Cordeliers, 15 Rue de l’École de Médecine, 75006 Paris, France.; 3 Metabolomics and Cell Biology Platforms, Institut Gustave Roussy, Pavillon de Recherche 1, 94805 Villejuif, France.; 4 Université Paris Descartes, Sorbonne Paris Cité, 12 Rue de l’École de Médecine, 75006 Paris, France.; 5 Pôle de Biologie, Hôpital Européen Georges Pompidou, AP-HP, 20 Rue Leblanc, 75015 Paris, France.

**Keywords:** hormesis, stress resistance, aging, neurodegeneration, therapeutic preconditioning

## Abstract

All living organisms need to adapt to ever changing adverse conditions in order to survive. The phenomenon termed hormesis describes an evolutionarily conserved process by which a cell or an entire organism can be preconditioned, meaning that previous exposure to low doses of an insult protects against a higher, normally harmful or lethal dose of the same stressor. Growing evidence suggests that hormesis is directly linked to an organism’s (or cell’s) capability to cope with pathological conditions such as aging and age-related diseases. Here, we condense the conceptual and potentially therapeutic importance of hormesis by providing a short overview of current evidence in favor of the cytoprotective impact of hormesis, as well as of its underlying molecular mechanisms.

In his masterpiece "The Twilight of the Idols", the German philosopher Friedrich Nietzsche stated: "What does not kill me, makes me stronger". Indeed, at least in specific cases, exposure to low amounts of a toxic substance or stressor may render an organism more resistant to higher (and otherwise detrimental) doses of the same trigger. This adaptive response is known as hormesis (from the Greek "to set in motion"): the exposure to mild levels of harmful factors preconditions a cell or an organism in that it stimulates the activation of stress resistance mechanisms, thus fostering the cellular capacity of maintenance and repair. Accordingly, in toxicology, hormesis defines a two-phase dose-response relationship, in which a low and a high dose trigger a stimulatory (beneficial) and an inhibitory (toxic) effect, respectively 1. Thus, mild and periodic (but not severe or chronic) exposure to specific factors characteristic for detrimental conditions such as aging and associated diseases should improve an organism’s ability to stably cope with such adverse circumstances. In this short review, we summarize evidence in favor of hormetic features in improved resistance against acute and long-term stress.

The universal and deep-seated concept of hormesis can be exemplified in a variety of different experimental models. For example, treatment of bacteria with sub-lethal concentrations of antibiotics not only provokes the surge of resistant populations, but also induces a general stress response, such as increased biofilm formation that contributes to elevated adaptability 2. In yeast, this leitmotif of activated stress response pathways becomes detectable upon treatment with low doses of H_2_O_2, _rendering cells more resistant to subsequent exposure to higher H_2_O_2 _doses 3. The resistance to high H_2_O_2 _concentrations depends on the formation of low levels of superoxide anions, which presumably drive an adaptive program 4. Of note, low oxidative stress has also been implicated in lifespan extension by rapamycin 5. These adaptations can ultimately culminate in increased longevity. Accordingly, inactivation of the hydrogen peroxide-detoxifying enzyme catalase or inhibition of the production of the reactive oxygen species (ROS)-scavenger glutathione extends yeast lifespan 6.

The involvement of mild oxidative stress in the hormetic response has spotlighted mitochondria as central control levers for hormesis, coining the term "mitohormesis" 7. In the nematode *C. elegans*, for instance, moderate mitochondrial stress 8 as well as ROS stress induced by hyperbaric oxygen or treatment with juglon (a natural dye found in walnut leaves) 9 decelerates aging. Flies preconditioned by hypoxia or low-dose gamma irradiation are more resistant to subsequent irradiation or aging-induced oxidative damage 10. Of note, even when preconditioned only in young age, this alleviation is measurable in old flies 10. Consistently, animals held under short-time ischemic/hypoxic states ("ischemic preconditioning"), are less susceptible to damage by subsequent strokes 11 and mice chronically exposed to mild irradiation show an extended mean lifespan of up to 22% 12. To date, it is not fully understood how hormetic responses to diverse stresses mediate lifespan extension, but there are reasons to believe that more genetic or pharmacological anti-aging interventions than expected might carry out their beneficial effects through hormetic mechanisms. For instance, longevity extension by caloric restriction or rapamycin has been shown to induce stress response pathways, similar to hormetic preconditioning 13,14.

Mechanistically, hormesis seems to be executed by a variety of physiological cellular processes which (probably cooperatively) converge on enhanced stress resistance and longevity (Figure 1). For example, mild heat stress in flies leads to increased expression of stress response proteins, including heat shock proteins that are detectable after more than a week post-treatment 15. Another example: the levels of mitochondrial uncoupling protein 2 (UCP2), which has been attributed a protective role against cytokine-induced pancreatic β-cell death 16, are strikingly elevated after oxidative stress in β-cells for several weeks 17. Hormesis could also favor a euproteomic state via the unfolded protein response (UPR), as low doses of ER-stress trigger the activation of proteostasis networks 18. In fact, this mechanism might also be connected to hypoxic/ischemic preconditioning 19. There is also evidence that the induction of cytoprotective autophagy, for example by low levels of BH3-mimetics (e.g. ABT737) that liberate pro-autophagic Beclin 1 from Bcl-2 proteins, or treatment of mouse hepatocytes with the secondary plant metabolite zerumbone, could contribute to hormesis 20,21,22. Future studies should address how mitochondrial signaling, proteostatis networks and/or other cytoprotective responses contribute to hormesis and which molecular key players are causally involved in the beneficial aftermath of low-dose stresses.

**Figure 1:**
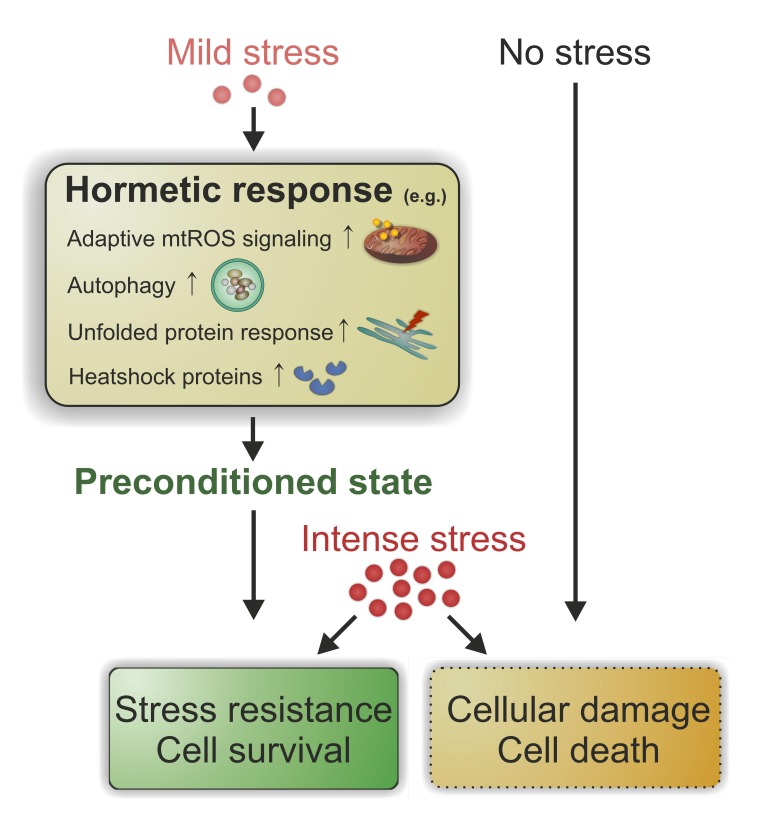
FIGURE 1: Hormesis governs a pleiotropic pro-survival program. When exposed to mild stress, cells/organisms respond by a variety of adaptive cellular programs that procure a preconditioned state. When an intense stress is applied subsequently, preconditioned but not naïve cells/organisms exhibit stress resistance and eventually improved survival. mtROS, mitochondrial reactive oxygen species.

The relevance of hormesis for both human pathophysiology and specific disease treatment is being increasingly recognized. As already mentioned, many anti-aging interventions may follow hormetic features, suggesting that preconditioning might have a preventive medical character. Accordingly, mild dietary stress, i.e. calorie restriction without malnutrition, as it can be achieved through different fasting regimens 23, may exert its beneficial effects on life- and healthspan, at least in part, through hormetic mechanisms 24. Along the same lines, exercise may counteract aging by virtue of a hormetic dose-response relationship. Thus, both lack of physical activity and overtraining are harmful, while regular but moderate exercise is beneficial, possibly through ROS-mediated preconditioning 25.

Both dietary factors and moderate exercise have been linked to improved brain health through hormesis 26. Indeed, recent data suggest an intriguing connection between beneficial low-dose responses and amyloid diseases. The accumulation of amyloid aggregates in the brain, which could exemplify a high-dose stress, represents one of the main hallmarks of these disorders. Strikingly, the exposure to non-harmful low doses of amyloid aggregates exerts a protective stimulus in models of Alzheimer’s and Parkinson’s disease 27,28,29. Similarly, a recent study suggests that the exposure of pancreatic β-cells to subtoxic concentrations of human islet amyloid polypeptide (hIAPP) aggregates may protect them through the hormetic stimulation of an antioxidant response 30. The accumulation of hIAPP aggregates in the islets of Langerhans is connected to pancreatic β-cell deterioration, a crucial characteristic of type 2 diabetes. Of note, hormesis has been also related to exercise-mediated protection against diabetes and the resistance to lifestyle factors that promote diabetes 31.

Besides these preventive features, hormesis has a direct clinical relevance. For instance, the ischemic preconditioning frequently used before heart surgery, where short and mild cycles of ischemia are applied, protects the heart and brain against the subsequent, more prolonged deprivation of oxygen and nutrients 21. In the field of drug development and patient treatment, the consideration that a given agent might induce hormetic dose-responses can yield useful results at two levels. First, when protective effects constitute the therapeutic goal, a small dose of a hormetic agent may be more useful than a high (close-to-toxic) dose. For instance, hormetic dose-responses might be crucial for drug-enhanced memory improvement in Alzheimer’s disease patients 32. Second, when destructive effects are desired, it should be avoided to underdose the therapeutic effect. For instance, the sublethal application of antibiotics can result in multidrug resistance and thus the perseverance of pathogenic micrororgansisms, possibly through ROS-induced mutagenesis 33. In the context of anticancer therapies, underdosing might favor the persistence of neoplastic cells in the organism. Moreover, the progressive degradation of an administered drug might result in hormetically active low doses, especially if an agent has a very long half-life 32, thus resulting in a similar outcome.

To sum up, hormesis defines a biphasic dose-response relationship that has a direct impact on preventive and clinical medicine. While the main molecular trait determining the beneficial character of low-dose stimulation for a cell or organism seems to be rooted in the activation of stress resistance, the detailed mechanisms underlying this phenomenon still need to be elucidated. In the same lines, the pleiotropic impact of hormesis on the human body must be explored and evaluated in further detail. Irrespectively, accumulating evidence concedes a point to Nietzsche’s thoughts: a little stress for some more stamina.
